# A population-based longitudinal study on glycated hemoglobin levels and new-onset chronic kidney disease among non-diabetic Japanese adults

**DOI:** 10.1038/s41598-023-40300-8

**Published:** 2023-08-23

**Authors:** Yukari Okawa, Etsuji Suzuki, Toshiharu Mitsuhashi, Toshihide Tsuda, Takashi Yorifuji

**Affiliations:** 1 Department of Epidemiology, Graduate School of Medicine, Dentistry and Pharmaceutical Sciences, Okayama University, 2-5-1 Shikata-Cho, Kita-Ku, Okayama, 700-8558 Japan; 2 Department of Epidemiology, Harvard T.H. Chan School of Public Health, Boston, MA USA; 3 Center for Innovative Clinical Medicine, Okayama University Hospital, Okayama, Japan; 4 Department of Human Ecology, Graduate School of Environmental and Life Science, Okayama University, Okayama, Japan

**Keywords:** Public health, Epidemiology, Population screening

## Abstract

Chronic kidney disease (CKD) is a major global public health problem. Recent studies reported that diabetes and prediabetes are risk factors for developing CKD; however, the exact glycated hemoglobin (HbA1c) cut-off value for prediabetes remains controversial. In this study, we aimed to examine the relationship between HbA1c levels and subsequent CKD development in greater detail than previous studies. Longitudinal data of annual checkups of 7176 Japanese non-diabetic people (male: 40.4%) from 1998 to 2022 was analyzed. HbA1c values were categorized into < 5.0%, 5.0–5.4%, 5.5–5.9%, and 6.0–6.4%. CKD was defined as an estimated glomerular filtration rate < 60 ml/min/1.73 m^2^. The descriptive statistics at study entry showed that higher HbA1c values were associated with male, older, overweight or obese, hypertensive, or dyslipidemic people. During a mean follow-up of 7.75 person-years, 2374 participants (male: 40.0%) developed CKD. The Weibull accelerated failure time model was selected because the proportional hazards assumption was violated. The adjusted time ratios of developing CKD for HbA1c levels of 5.5–5.9% and 6.0–6.4% compared with 5.0–5.4% were 0.97 (95% confidence interval: 0.92–1.03) and 1.01 (95% confidence interval: 0.90–1.13), respectively. There was no association between HbA1c in the prediabetic range and subsequent CKD development.

## Introduction

Chronic kidney disease (CKD) has been increasing globally and is a major public health problem^1^. According to the Kidney Disease: Improving Global Outcomes, CKD is defined as abnormalities of kidney structure or function, persisting for more than 3 months, with implications for health^2^. Clinically, CKD is asymptomatic in its early stages and tends to progress without being noticed. Once kidney function is reduced during the development of end-stage renal disease, advanced treatment options such as renal replacement therapies are necessary. Unfortunately however, such treatment has significant drawbacks, including a long waiting period before receiving a transplant, limited availability due to cost, particularly in low-income countries, and that its initiation itself is associated with an increased risk for mortality^3–6^. Given that even early stages of CKD are a risk factor for stroke, cardiovascular disease, hospitalization, and mortality, prevention of new-onset CKD is crucial^7–9^.

Previous studies reported a variety of risk factors for developing CKD, including male, older age, non-Hispanic black, obesity, cigarette smoking, hypertension, metabolic syndrome, diabetes, hyperuricemia, Western-type dietary pattern, low level of physically activity, and low socioeconomic status^10–20^. Among these, diabetes, which affects 10.6% of the global adult population, is a major factor in CKD development^16,21^.

In 2016, a meta-analysis of nine cohort studies from six countries reported that the overall risk of developing CKD was 1.11 times higher (95% confidence interval (CI): 1.02–1.21), even for participants with prediabetes^22^. Unfortunately, the study used multiple definitions of prediabetes with different indicators, including fasting plasma glucose, 2-h plasma glucose, and glycated hemoglobin (HbA1c). Among these, HbA1c is officially used as an indicator of glycemic control status because it is globally standardized, has low intrasubject variability, reflects the glycemic control of the previous 120 days, and is unaffected by dietary intake^23^.

In a more recent meta-analysis of the relationship between prediabetes and CKD development, the authors used HbA1c as an indicator of prediabetes: HbA1c 5.7–6.4% (< 5.7% as reference) as recommended by the American Diabetes Association (ADA) and HbA1c 6.0–6.4% (< 6.0% as reference) recommended by the International Expert Committee (IEC) and the National Institute for Health and Clinical Excellence (NICE)^23–26^. Both HbA1c-defined prediabetes classifications showed similar results to those of previous meta-analyses^22,24^. However, while the ADA classification was used in three studies, the IEC/NICE classifications were used in only one study. Given this limited number of previous studies, additional research to assess the risk of developing CKD by the HbA1c level in more detail is of importance. Furthermore, it is also important to investigate the finer HbA1c cut-off value in the prediabetic range that increases the risk for developing CKD because it can be more efficiently used for prevention and early intervention of CKD.

Accordingly, we aimed to examine in more detail the relationship between HbA1c levels and subsequent CKD development in a non-diabetic Japanese population.

## Methods

### Data sources

This open cohort study used administrative data from the database of Zentsuji city, Kagawa Prefecture, Japan. The city is located in the central part of the Kagawa Prefecture with a population of 30,734 on June 1, 2022 (male: 49.9%)^27^.

All Zentsuji citizens aged ≥ 40 years by fiscal year were eligible for an annual health checkup, following the protocol specified by the Japanese Ministry of Health, Labour and Welfare. Additionally, the city conducted a checkup for younger citizens, aged 35–39 years by fiscal year age, following the same protocol. The protocol details are described elsewhere^28,29^.

The checkup included anthropometric measurements, a blood pressure test, a blood test, a urine dipstick test, and a self-administered questionnaire to identify the modifiable lifestyle factors. On average, 30–40% of the target population completed the checkup.

### Study population

All participants who underwent a checkup from April 1998 to June 2022 were included in this analysis. A total of 15,244 participants (male: 40.5%) agreed to participate in this study. Delayed entry was allowed in this analysis.

**Figure 1:**
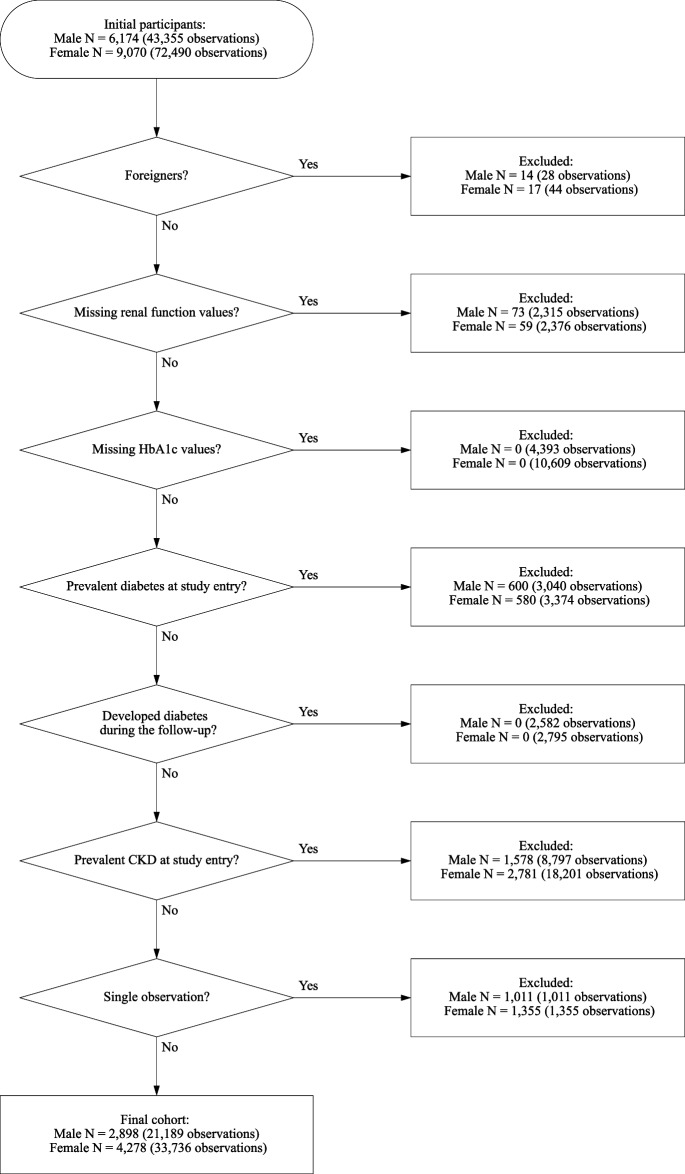
Participant flow chart of the study cohort.

To evaluate the relationship between HbA1c levels and subsequent CKD incidence in a non-diabetic Japanese population, the following exclusion criteria were applied: (a) foreigner, (b) missing renal function values, (c) prevalent CKD at study entry, (d) missing HbA1c values, (e) prevalent diabetes at study entry, and (f) single observation (Fig. 1). Participants who developed diabetes during the follow-up were censored.

### Measures

As an exposure variable, HbA1c was used as an indicator of blood glucose levels. HbA1c was reported in Japan Diabetes Society (JDS) units (%) from 1998 to 2012 and in National Glycohemoglobin Standardization Program (NGSP) units (%) from 2013 to 2022. HbA1c was standardized to NGSP values by the officially certified equation^30,31^:

1 $$HbA1c_{NGSP} \left( \% \right) = 1.02 \times HbA1c_{JDS} + 0.25.$$

HbA1c was categorized into four groups: < 5.0%, 5.0–5.4% (reference), 5.5–5.9%, and 6.0–6.4%. According to the ADA criteria, an HbA1c level ≥ 6.5% was regarded as prevalent diabetes^23^.

The outcome of interest was new-onset CKD. Following the 2013 Kidney Disease: Improving Global Outcomes guidelines, the estimated glomerular filtration rate (eGFR) was used as an overall index of renal function. A decreased eGFR to < 60 mL/min/1.73 m^2^ (eGFR categories G3a–G5) was regarded as prevalent CKD^2^. The eGFR was calculated by the revised Japanese equation^2,32^:

2 $$eGFR_{Japanese} \left( {{\text{mL}}/\min/1.73{\text{m}}^{2} } \right) = 194 \times serum\;creatinine ^{ - 1.094} \times age^{ - 0.287} \times 0.739 \left( { if\;female} \right).$$

Serum creatinine was measured in mg/dL to two decimal places, using enzymatic methods.

**Figure 2:**
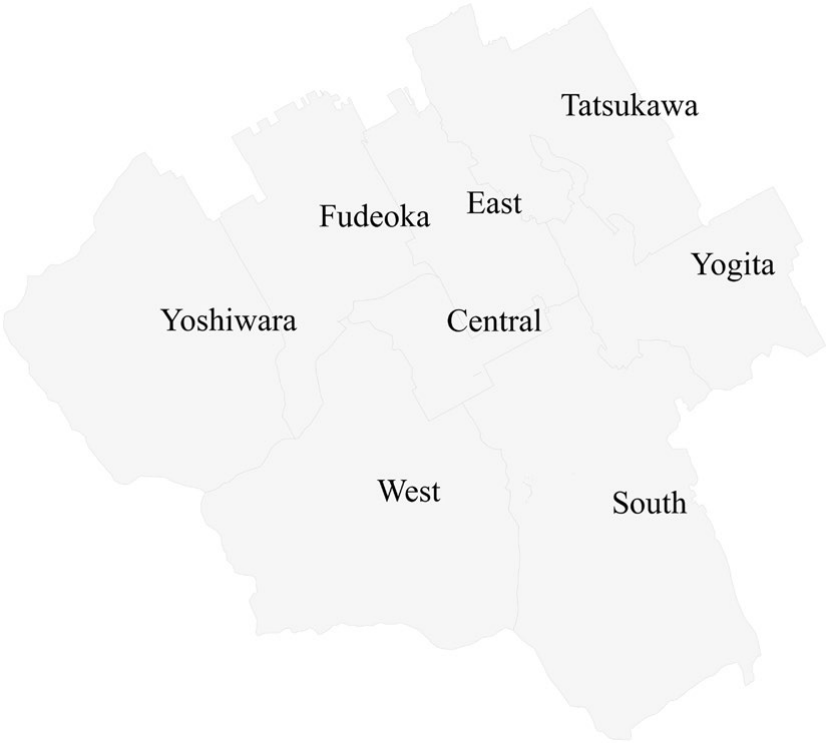
Map of the Zentsuji city districts.

To reduce potential bias, socio-demographic and modifiable lifestyle factors were adjusted. Socio-demographic variables included age, sex, and residential area. Age was coded in three groups: 34–59, 60–69, and ≥ 70 years. Sex was coded in male and female. Residential area was categorized into the following eight districts: East, West, Central, South, Fudeoka, Tatsukawa, Yogita, and Yoshiwara (Fig. 2).

Modifiable lifestyle factors included the body mass index (BMI), self-reported smoking status, self-reported drinking status, hypertension, and dyslipidemia. The BMI was calculated as the weight in kilograms over the height in meters squared. On the basis of the World Health Organization recommended BMI classification, BMI was dichotomized as “overweight or obese” (BMI ≥ 25 kg/m^2^) or otherwise^33^. Self-reported smoking status was coded in “non- or ex-smoker” and “current smoker.” Self-reported drinking status was categorized as “non- or seldom-drinker” and “drinker.” Hypertension was defined as a systolic blood pressure ≥ 140 mmHg and/or a diastolic blood pressure ≥ 90 mmHg^34^. Dyslipidemia was defined as serum low-density lipoprotein cholesterol ≥ 140 mg/dL and/or serum high-density lipoprotein cholesterol < 40 mg/dL^35^.

### Statistical analysis

Characteristics at study entry were summarized according to the HbA1c categories. Continuous variables were expressed as the mean and standard deviation or median and the interquartile range (IQR). Categorical variables were summarized by counts and proportions. The number of observations per participant during the follow-up period was summarized as the mean, standard deviation, median, IQR, count, and proportion and stratified by event occurence.

**Figure 3:**
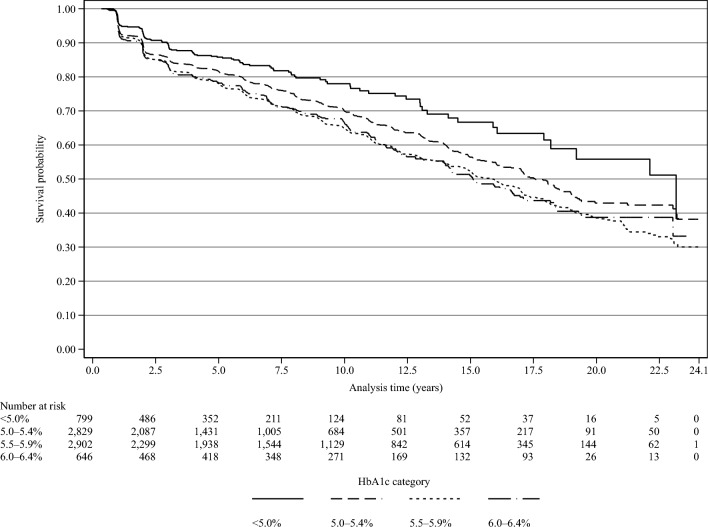
Kaplan–Meier survival estimates by HbA1c category.

The person-year at risk was calculated from the date of the first checkup to the occurrence of an event or the onset of diabetes, otherwise to the end of the last observation during follow-up. The Kaplan–Meier curves were drawn in Fig. 3. The proportional hazards (PH) assumption across the HbA1c categories was assessed using both graphical methods and statistical test^36^. Because the data displayed a nonconstant hazard function and all the covariates except sex were time-varying, we decided to use the Weibull accelerated failure time model. The models were fitted based on the Akaike and Bayesian information criterions^37,38^. The estimates were shown in adjusted time ratios (aTRs) and 95% CIs. A time ratio > 1 means a longer survival time due to covariate effects, whereas a time ratio ≤ 1 means the same or shorter survival time.

Covariate selection was performed based on previous findings^39,40^. To assess the presence of an interaction effect, we added an interaction term between the HbA1c category and the following variables to Models 1–4: sex, age category, overweight or obese, self-reported drinking status, self-reported smoking status, hypertension, and dyslipidemia. A product term was added to models when we detected an interaction effect. Model 1 was adjusted for sex and age category, Model 2 was adjusted for both variables of Model 1 and overweight or obese, self-reported drinking status, and self-reported smoking status, Model 3 was adjusted for all variables of Model 2, hypertension, and dyslipidemia, and Model 4 was adjusted for all variables of Model 3 and residential area. Because the results for the crude and adjusted models were similar, only the results for Models 1–4 were presented in this analysis.

To handle missing data, the following variables were fulfilled by multiple imputation using chained equations: overweight or obese (missing 0.01%), hypertension (missing 0.01%), dyslipidemia (missing 2.79%), self-reported drinking status (missing 28.3%), self-reported smoking status (missing 26.4%), and residential area (missing 0.80%). Binary variables were imputed using logistic regression and categorical variables was imputed using multinominal logistic regression. Forty imputations were performed to meet the required number of imputations by Stata module “how_many_imputations”^41^. Self-reported smoking status and self-reported drinking status were likely to be missing not at random due to the administrative data collection circumstances. In this analysis, the results of multiple imputation were selected because these results and those of complete case analysis were similar and it was important to maintain statistical power^42^.

We performed six sensitivity analyses. In the first sensitivity analysis, we used the ADA classification of HbA1c and compared the results with those of a previous study: < 5.7% (reference) and 5.7–6.4%^24^. In the second sensitivity analysis, we further used the IEC/NICE classifications of HbA1c: < 6.0% (reference) and 6.0–6.4%^39^. In the third sensitivity analysis, a subgroup analysis was conducted to examine how the results differed by age category: 34–69 vesus ≥ 70 years. In the fourth sensitivity analysis, we excluded the following participants to minimize potential reverse causation: missing data of a urine dipstick test or proteinuria ≥  + 1 at study entry. In the fifth sensitivity analysis, we compared the results for the periods when HbA1c was reported in JDS units (1998–2012) and NGSP units (2013–2022)^30^. Finally, the sixth sensitivity analysis further investigated how the GFR estimation method affected the results by using the 2009 CKD Epidemiology Collaboration (CKD-EPI) equation (Eq. 3), which was recommended by the 2012 KDIGO guideline, and the 2009 CKD-EPI equation modified for the Japanese (Eq. 4)^2,43,44^:

3 $$\begin{aligned} eGFR_{CKD - EPI - 2009} \left( {{\text{mL}}/\min/1.73{\text{m}}^{2} } \right) & = 141 \times \min \left( {SCr/\kappa , 1} \right)^{\alpha } \times \max \left( {SCr/\kappa , 1} \right)^{ - 1.209} \times 0.993^{age} \\ \times 1.018 \left( {if\;female} \right) \times 1.159 \left( {if\;black} \right), \\ \end{aligned}$$

*wℎere*: κ = 0.9 (*male*) *or* 0.7 (*female*), α =  − 0.411 (*male*) *or* − 0.329 (*female*), *min is t*ℎ*e minimum of SCr*⁄κ*or* 1, *and max is t*ℎ*e maximum of SCr*⁄κ*or* 1.

4 $$\begin{gathered} eGFR_{CKD - EPI - 2009 - Japanese} \left( {{\text{mL}}/\min/1.73{\text{m}}^{2} } \right) \hfill \\ = 141 \times \min \left( {SCr/\kappa , 1} \right)^{\alpha } \times \max \left( {SCr/\kappa , 1} \right)^{ - 1.209} \times 0.993^{age} \times 1.018 \left( {if\;female} \right) \hfill \\ \times 1.159 \left( {if\;black} \right) \times 0.813 \left( {if\;Japanese} \right), \hfill \\ \end{gathered}$$

*where*: κ = 0.9 (*male*) *or* 0.7 (*female*), α =  − 0.411 (*male*) *or* − 0.329 (*female*), *min is t*ℎ*e minimum of SCr*⁄κ*or* 1, *and max is t*ℎ*e maximum of SCr*⁄κ*or* 1.

A two-tail *p*-value of < 0.05 was considered to be statistically significant. All statistical analyses were performed using STATA/SE 18.0 (StataCorp, College Station, Texas, USA). The Kaplan–Meier curves were drawn using the STATA “sts graph” command (Fig. 3). The geographic data were downloaded from the National Statistics Center website^45^. The participant flow (Fig. 1) and the district map of Zentsuji city (Fig. 2) were drawn in Python 3.10.11. The former was drawn using the “SchemDraw 0.16” package and the latter by the “GeoPandas 0.13.2” package^46,47^. Data analysis was conducted from March 2022 to July 2023. This study followed the Strengthening the Reporting of Observational Studies in Epidemiology reporting guidelines^48^.

### Ethics

All data were retrieved from the administrative database and anonymized prior to receipt. The Ethics Committee of Okayama University Graduate School of Medicine, Dentistry and Pharmaceutical Sciences and Okayama University Hospital approved this study and waived the need for informed consent (No. K1708-040). Study procedures were performed in accordance with the Declaration of Helsinki.

## Results

Of 15,244 initial participants (male: 40.5%) who completed the checkup, 7176 (male: 40.4%) remained in the final cohort. During a mean follow-up of 7.75 person-years and a median of 6.11 person-years, 2374 participants (male: 40.0%) experienced new-onset CKD. The group that developed CKD during the follow-up period had fewer observations per participant than the group that did not (Table 1).

**Table 1:** Summary of the number of observations during the follow-up period for 7176 non-diabetic Japanese citizens of Zentsuji city stratified by event occurrence (1998–2022).

Number of observations per participant	New-onset CKD during the follow-up				Total	
	No		Yes			
	N	(%)	N	(%)	N	(%)
2	949	(19.76%)	804	(33.87%)	1753	(24.43%)
3	618	(12.87%)	407	(17.14%)	1025	(14.28%)
4	524	(10.91%)	212	(8.93%)	736	(10.26%)
5	412	(8.58%)	143	(6.02%)	555	(7.73%)
6	349	(7.27%)	146	(6.15%)	495	(6.90%)
7	327	(6.81%)	146	(6.15%)	473	(6.59%)
8	261	(5.44%)	112	(4.72%)	373	(5.20%)
9	231	(4.81%)	90	(3.79%)	321	(4.47%)
10	262	(5.46%)	69	(2.91%)	331	(4.61%)
11	165	(3.44%)	70	(2.95%)	235	(3.27%)
12	129	(2.69%)	37	(1.56%)	166	(2.31%)
13	106	(2.21%)	32	(1.35%)	138	(1.92%)
14	88	(1.83%)	29	(1.22%)	117	(1.63%)
15	64	(1.33%)	13	(0.55%)	77	(1.07%)
16	77	(1.60%)	18	(0.76%)	95	(1.32%)
17	76	(1.58%)	15	(0.63%)	91	(1.27%)
18	52	(1.08%)	19	(0.80%)	71	(0.99%)
19	49	(1.02%)	7	(0.29%)	56	(0.78%)
20	48	(1.00%)	3	(0.13%)	51	(0.71%)
21	11	(0.23%)	2	(0.08%)	13	(0.18%)
22	2	(0.04%)	0	(0.00%)	2	(0.03%)
23	2	(0.04%)	0	(0.00%)	2	(0.03%)
Total	4802	(66.92%)	2374	(33.08%)	7176	(100%)
Mean (standard deviation)	6.60	(4.55)	5.07	(3.79)	6.09	(4.38)
Median (interquartile range)	5	(3–9)	3	(2–7)	5	(3–8)

In Table 2, we present characteristics of the 7176 participants at study entry stratified according to HbA1c levels. Of the participants without diabetes, 31.4% (male: 36.2%) exhibited CKD at study entry and were excluded from the analysis (Fig. 1). The proportions of non-diabetic participants with CKD at study entry by age category were 14.6% in the 34–59 age category, 28.8% in the 60–69 category, and 51.3% in the ≥ 70 age category. In the final cohort, those participants with higher HbA1c levels were more likely to be male, older, overweight or obese, hypertensive, or dyslipidemic at study entry. In all the HbA1c groups, approximately 50% of the participants lived in either the East, Tatsukawa, or South areas.

**Table 2:** Descriptive characteristics of the first observation of 7176 non-diabetic Japanese citizens of Zentsuji city stratified by HbA1c category at study entry.

	HbA1c category									
	< 5.0%		5.0–5.4%		5.5–5.9%		6.0–6.4%		Total	
	N	(%)	N	(%)	N	(%)	N	(%)	N	(%)
Total	799	(11.13%)	2829	(39.42%)	2902	(40.44%)	646	(9.00%)	7176	(100%)
Variables										
Sex										
Female	484	(60.58%)	1704	(60.23%)	1752	(60.37%)	338	(52.32%)	4278	(59.62%)
Male	315	(39.42%)	1125	(39.77%)	1150	(39.63%)	308	(47.68%)	2898	(40.38%)
Age category										
34–59	437	(54.69%)	1276	(45.10%)	1013	(34.91%)	176	(27.24%)	2902	(40.44%)
60–69	223	(27.91%)	973	(34.39%)	1224	(42.18%)	301	(46.59%)	2721	(37.92%)
≥ 70	139	(17.40%)	580	(20.50%)	665	(22.92%)	169	(26.16%)	1553	(21.64%)
BMI category										
Normal	658	(82.35%)	2273	(80.35%)	2077	(71.57%)	400	(61.92%)	5408	(75.36%)
Overweight or obese^a^	141	(17.65%)	556	(19.65%)	824	(28.39%)	246	(38.08%)	1767	(24.62%)
Missing	0	(0.00%)	0	(0.00%)	1	(0.03%)	0	(0.00%)	1	(0.01%)
Self-reported drinking status										
Nondrinker	297	(37.17%)	1064	(37.61%)	1155	(39.80%)	284	(43.96%)	2800	(39.02%)
Drinker	259	(32.42%)	829	(29.30%)	730	(25.16%)	157	(24.30%)	1975	(27.52%)
No answer	243	(30.41%)	936	(33.09%)	1017	(35.04%)	205	(31.73%)	2401	(33.46%)
Self-reported smoking status										
Nonsmoker	433	(54.19%)	1490	(52.67%)	1557	(53.65%)	361	(55.88%)	3841	(53.53%)
Smoker	130	(16.27%)	424	(14.99%)	349	(12.03%)	85	(13.16%)	988	(13.77%)
No answer	236	(29.54%)	915	(32.34%)	996	(34.32%)	200	(30.96%)	2347	(32.71%)
Hypertension^b^										
No	574	(71.84%)	1984	(70.13%)	2012	(69.33%)	397	(61.46%)	4967	(69.22%)
Yes	225	(28.16%)	844	(29.83%)	889	(30.63%)	249	(38.54%)	2207	(30.76%)
Missing	0	(0.00%)	1	(0.04%)	1	(0.03%)	0	(0.00%)	2	(0.03%)
Dyslipidemia^c^										
No	547	(68.46%)	1813	(64.09%)	1636	(56.37%)	322	(49.85%)	4318	(60.17%)
Yes	211	(26.41%)	889	(31.42%)	1138	(39.21%)	283	(43.81%)	2521	(35.13%)
Missing	41	(5.13%)	127	(4.49%)	128	(4.41%)	41	(6.35%)	337	(4.70%)
Residential area										
East	137	(17.15%)	534	(18.88%)	530	(18.26%)	118	(18.27%)	1319	(18.38%)
Tatsukawa	116	(14.52%)	453	(16.01%)	506	(17.44%)	111	(17.18%)	1186	(16.53%)
South	107	(13.39%)	406	(14.35%)	450	(15.51%)	124	(19.20%)	1087	(15.15%)
Fudeoka	126	(15.77%)	327	(11.56%)	353	(12.16%)	82	(12.69%)	888	(12.37%)
Center	106	(13.27%)	311	(10.99%)	294	(10.13%)	51	(7.89%)	762	(10.62%)
Yoshiwara	83	(10.39%)	281	(9.93%)	263	(9.06%)	46	(7.12%)	673	(9.38%)
West	60	(7.51%)	271	(9.58%)	261	(8.99%)	68	(10.53%)	660	(9.20%)
Yogita	51	(6.38%)	218	(7.71%)	208	(7.17%)	42	(6.50%)	519	(7.23%)
Missing	13	(1.63%)	28	(0.99%)	37	(1.27%)	4	(0.62%)	82	(1.14%)

Abbreviations: BMI, body mass index; HbA1c, glycated hemoglobin.

^a^Overweight or obese is defined as a BMI ≥ 25 kg/m^2^.

^b^Hypertension is defined as systolic blood pressure ≥ 140 mmHg and/or diastolic blood pressure ≥ 90 mmHg.

^c^Dyslipidemia is defined as serum low density lipoprotein cholesterol ≥ 140 mg/dL and/or serum high density lipoprotein cholesterol < 40 mg/dL.

Table 3 shows descriptive statistics of the distribution of the 7176 participants stratified by HbA1c levels during the follow-up period with missing values completed by multiple imputation methods. Results for multiple imputed variables (overweight or obese, self-reported drinking status, self-reported smoking status, hypertension, dyslipidemia, and residential area) were averaged over 40 imputations. The HbA1c < 5.0% and HbA1c 6.0–6.4% groups had a slightly shorter follow-up period than the other groups. Kaplan–Meier curves showed that the HbA1c < 5.0% group had a higher survival probability compared with the other HbA1c categories (Fig. 3).

**Table 3:** Descriptive statistics of all observations of 7176 non-diabetic Japanese citizens of Zentsuji city stratified by HbA1c category with missing values completed by multiple imputation method (1998–2022).

	HbA1c category														
	< 5.0%			5.0–5.4%			5.5–5.9%			6.0–6.4%			Total		
	Failure	PY	IR*	Failure	PY	IR*	Failure	PY	IR*	Failure	PY	IR*	Failure	PY	IR*
Time at risk															
Total		4441.2			19,532.1			26,026.6			5604.5			55,604.4	
Minimum		0.63			0.48			0.34			0.54			0.34	
Maximum		22.7			23.9			24.0			21.3			24.1	
Mean		3.76			4.76			5.44			3.66			7.75	
Median		2.08			3.10			4.01			2.13			6.11	
Failure	129			779			1210			256			2374		
IR^a^			29.0			39.9			46.5			45.7			42.7
Variables															
Sex															
Female	63	2743.3	23.0	471	12,051.7	39.1	722	16,286.7	44.3	169	3499.7	48.3	1425	34,581.4	41.2
Male	66	1697.9	38.9	308	7480.4	41.2	488	9739.9	50.1	87	2104.8	41.3	949	21,023.0	45.1
Age category															
34–59	28	2516.1	11.1	174	7770.6	22.4	197	7204.5	27.3	28	1062.6	26.4	427	18,553.8	23.0
60–69	40	1009.9	39.6	235	6223.7	37.8	409	9793.1	41.8	95	2302.9	41.3	779	19,329.6	40.3
≥ 70	61	915.2	66.7	370	5537.8	66.8	604	9029.0	66.9	133	2239.0	59.4	1168	17,721.0	65.9
BMI category															
Normal	100.0	3727.2	26.8	608.0	15,893.8	38.3	855.0	19,587.1	43.7	159.0	3660.6	43.4	1722.0	42,868.7	40.2
Overweight or obese^b^	29.0	714.0	40.6	171.0	3638.3	47.0	355.0	6439.5	55.1	97.0	1943.9	49.9	652.0	12,735.7	51.2
Self-reported drinking status															
Nondrinker	62.7	2338.2	26.8	478.9	11,171.6	42.9	769.2	15,849.7	48.5	171.0	3575.9	47.8	1481.7	32,935.4	45.0
Drinker	66.4	2103.0	31.5	300.1	8360.5	35.9	440.8	10,176.9	43.3	85.0	2028.6	41.9	892.3	22,669.0	39.4
Self-reported smoking status															
Nonsmoker	104.3	3416.6	30.5	658.7	15,816.1	41.6	1022.6	21,799.5	46.9	219.2	4741.9	46.2	2004.8	45,774.1	43.8
Smoker	24.8	1024.6	24.2	120.3	3716.0	32.4	187.4	4227.0	44.3	36.8	862.6	42.6	369.2	9830.3	37.6
Hypertension^c^															
No	74.0	3210.6	23.0	481.0	14,210.5	33.8	795.0	18,481.4	43.0	140.0	3766.8	37.2	1490.0	39,669.2	37.6
Yes	55.0	1230.6	44.7	298.0	5321.6	56.0	415.0	7545.2	55.0	116.0	1837.7	63.1	884.0	15,935.1	55.5
Dyslipidemia^d^															
No	86.8	3243.2	26.7	531.2	13,480.2	39.4	760.4	16,156.2	47.1	132.9	3144.1	42.3	1511.2	36,023.8	41.9
Yes	42.3	1198.0	35.3	247.8	6051.9	41.0	449.6	9870.4	45.6	123.2	2460.4	50.1	862.9	19,580.6	44.1
Residential area															
East	20.5	851.1	24.0	151.1	3649.0	41.4	245.5	5122.4	47.9	59.6	1178.3	50.5	476.6	10,800.8	44.1
Tatsukawa	13.3	697.6	19.1	116.7	3022.2	38.6	183.0	4637.6	39.4	38.4	959.9	40.0	351.4	9317.3	37.7
South	22.5	620.7	36.3	97.9	2879.8	34.0	186.4	4059.6	45.9	46.4	888.7	52.2	353.2	8448.8	41.8
Fudeoka	16.3	647.2	25.1	94.3	2459.2	38.4	141.4	3020.4	46.8	28.6	713.3	40.0	280.5	6840.1	41.0
Center	25.1	557.9	45.0	109.0	2244.4	48.6	126.7	2538.5	49.9	25.4	443.3	57.2	286.2	5784.1	49.5
Yoshiwara	11.2	387.4	28.9	90.2	2035.1	44.3	119.8	2397.9	50.0	20.3	498.5	40.7	241.4	5318.9	45.4
West	11.1	384.4	28.9	70.1	1781.2	39.3	119.8	2344.9	51.1	22.3	548.6	40.7	223.3	5059.1	44.1
Yogita	9.1	294.9	30.7	49.8	1461.2	34.0	87.6	1905.3	46.0	15.2	373.9	40.5	161.5	4035.3	40.0

Results for multiple imputed variables (overweight or obese, self-reported drinking status, self-reported smoking status, hypertension, dyslipidemia, and residential area) are averaged over 40 imputations.

BMI, Body mass index; HbA1c, Glycated hemoglobin; IR, Incidence rate; PY, Person-years.

^a^Incidence rate is reported per 1000 person-years.

^b^Overweight or obese is defined as a BMI ≥ 25 kg/m^2^.

^c^Hypertension is defined as systolic blood pressure ≥ 140 mmHg and/or diastolic blood pressure ≥ 90 mmHg.

^d^Dyslipidemia is defined as serum low density lipoprotein cholesterol ≥ 140 mg/dL and/or serum high density lipoprotein cholesterol < 40 mg/dL.

Table 4 shows the aTRs for CKD occurrence, with missing values filled by multiple imputation methods. In all models, there was an interaction between HbA1c < 5.0% and age category. With HbA1c 5.0–5.4% as the reference group, the point estimates of the aTR for developing CKD were slightly lower in the prediabetic range of HbA1c 5.5–5.4% and slightly higher in the prediabetic range of HbA1c 6.0–6.4%. However, the 95% CI of all HbA1c groups in the prediabetic range was across null in all models. Among all the HbA1c groups, the HbA1c < 5.0% group had the highest point estimate of a 20% elevated aTR (95% CI 7–35%) in the fully adjusted model.

**Table 4:** New onset of chronic kidney disease by HbA1c category among 7176 non-diabetic Japanese citizens of Zentsuji city (Total time at risk: 55,604.4 person-years, 1998–2022).

Covariates	Model 1	Model 2	Model 3	Model 4
	aTR (95% CI)	aTR (95% CI)	aTR (95% CI)	aTR (95% CI)
HbA1c category				
< 5.0%	1.20 (1.07–1.34)	1.20 (1.07–1.34)	1.20 (1.07–1.35)	1.20 (1.07–1.35)
5.0–5.4% (reference)	1.00	1.00	1.00	1.00
5.5–5.9%	0.97 (0.92–1.03)	0.97 (0.92–1.03)	0.97 (0.92–1.03)	0.97 (0.92–1.03)
6.0–6.4%	1.00 (0.90–1.12)	1.01 (0.91–1.13)	1.01 (0.91–1.13)	1.01 (0.90–1.13)
Sex				
Female (reference)	1.00	1.00	1.00	1.00
Male	1.00 (0.98–1.02)	0.99 (0.97–1.02)	0.99 (0.96–1.02)	0.99 (0.96–1.02)
Age category				
34–59 (reference)	1.00	1.00	1.00	1.00
60–69	1.04 (0.98–1.10)	1.04 (0.98–1.10)	1.04 (0.98–1.10)	1.04 (0.98–1.10)
≥ 70	0.99 (0.93–1.07)	1.00 (0.93–1.07)	1.00 (0.93–1.07)	1.00 (0.93–1.07)
BMI category^a^				
Normal (reference)		1.00	1.00	1.00
Overweight or obese^a^		0.94 (0.92–0.96)	0.95 (0.92–0.97)	0.95 (0.92–0.97)
Self-reported drinking status				
Nondrinker (reference)		1.00	1.00	1.00
Drinker		1.01 (0.98–1.03)	1.01 (0.98–1.04)	1.01 (0.98–1.04)
Self-reported smoking status				
Nonsmoker (reference)		1.00	1.00	1.00
Smoker		1.01 (0.98–1.05)	1.01 (0.98–1.05)	1.01 (0.98–1.05)
Hypertension^b^				
No (reference)			1.00	1.00
Yes			0.95 (0.93–0.98)	0.95 (0.93–0.97)
Dyslipidemia^c^				
No (reference)			1.00	1.00
Yes			1.00 (0.97–1.02)	1.00 (0.97–1.02)
Residential area				
East (reference)				1.00
Tatsukawa				1.04 (1.00–1.08)
South				1.02 (0.98–1.06)
Fudeoka				1.01 (0.97–1.06)
Center				0.98 (0.94–1.02)
Yoshiwara				0.98 (0.94–1.03)
West				1.00 (0.96–1.05)
Yogita				1.05 (1.00–1.10)
Interaction				
HbA1c category × age category				
(reference: HbA1c 5.0–5.4%, 34–59 years)				
HbA1c < 5.0% × age 60–69	0.82 (0.71–0.95)	0.83 (0.71–0.96)	0.83 (0.71–0.96)	0.83 (0.71–0.96)
HbA1c < 5.0% × age ≥ 70	0.84 (0.73–0.96)	0.84 (0.73–0.96)	0.84 (0.73–0.96)	0.84 (0.73–0.96)
HbA1c 5.5–5.9% × age 60–69	1.00 (0.93–1.07)	1.00 (0.93–1.07)	1.00 (0.93–1.07)	1.00 (0.93–1.07)
HbA1c 5.5–5.9% × age ≥ 70	1.03 (0.96–1.10)	1.03 (0.96–1.10)	1.03 (0.96–1.10)	1.03 (0.96–1.10)
HbA1c 6.0–6.4% × age 60–69	0.97 (0.86–1.10)	0.97 (0.86–1.10)	0.97 (0.86–1.10)	0.97 (0.85–1.10)
HbA1c 6.0%–6.4% × age ≥ 70	1.02 (0.91–1.16)	1.03 (0.91–1.16)	1.03 (0.91–1.16)	1.03 (0.91–1.16)

Model 1: Adjusted for sex and age category.

Model 2: Adjusted for both variables of Model 1, overweight or obese, self-reported drinking status, and self-reported smoking status.

Model 3: Adjusted for all variables of Model 2, hypertension, and dyslipidemia.

Model 4: Adjusted for all variables of Model 3 and residential area.

aTR, adjusted time ratio; CI, Confidence interval; HbA1c, Glycated hemoglobin.

^a^Overweight or obese is defined as a body mass index ≥ 25 kg/m^2^.

^b^Hypertension is defined as systolic blood pressure ≥ 140 mmHg and/or diastolic blood pressure ≥ 90 mmHg.

^c^Dyslipidemia is defined as serum low density lipoprotein cholesterol ≥ 140 mg/dL and/or serum high density lipoprotein cholesterol < 40 mg/dL.

Multiple imputed variables: overweight or obese, self-reported drinking status, self-reported smoking status, hypertension, dyslipidemia, and residential area.

The results of the first and the second sensitivity analyses using the ADA and the IEC/NICE classifications of HbA1c are shown in Supplementary Tables S1 and S2, respectively. Both results displayed similar trends to the main analysis in Table 4, and we found no clear association between HbA1c in the prediabetic range and subsequent CKD development.

The results of subgroup analysis by age category for the third sensitivity analysis are shown in Supplementary Table S3. Total time at risk were 42.0% shorter in the 34–69 age category and 56.9% shorter in the ≥ 70 age category. Because of convergence errors in the results for the ≥ 70 age category, only results for the 34–69 age category are shown. In the full model, the point estimates of all HbA1c categories were slightly lower than those of the main analysis, which included older participants. All 95% CIs were across null in all models.

Supplementary Table S4 shows the results of the fourth sensitivity analysis minimizing reverse causation, and excluding participants with missing data of a urine dipstick test or with a proteinuria ≥  + 1 at study entry. The fourth sensitivity analysis showed a slightly longer time to CKD onset compared with those of the main analysis in Table 4.

Supplementary Table S5 shows the results of the fifth sensitivity analysis, comparing the period 1998–2012, when HbA1c was reported in JDS, with the period 2013–2022, when it was reported in NGSP units. The median HbA1c values in JDS and NGSP units were 5.1% (IQR 4.9–5.4%) and 5.6% (IQR 5.4–5.8%), respectively. The total time at risk was relatively shorter in the HbA1c 6.0–6.4% group (715.8 person-years, 2.67% of the total) for the JDS unit, and HbA1c < 5.0% group (593.5 person-years, 2.65% of the total) for the NGSP unit. For HbA1c reported in JDS units, prediabetes (HbA1c 5.5–5.9% and HbA1c 6.0–6.4%) displayed higher point estimates in all models compared with the main analysis in Table 4. For HbA1c reported in NGSP units, prediabetes diplayed slightly higher point estimates compared with the main analysis, with an observed interaction between HbA1c < 5.0% and age category. However, the results showed no clear association between HbA1c in the prediabetic range and CKD.

In the final sensitivity analysis, we further estimated the GFR using the the 2009 CKD-EPI equation (Eq. 3) and the 2009 CKD-EPI equation modified for the Japanese (Eq. 4). The median eGFR values were 90 mL/min/1.73 m^2^ (IQR 81–98 mL/min/1.73 m^2^) and 75 mL/min/1.73 m^2^ (IQR 68–81 mL/min/1.73 m^2^), respectively. The median eGFR using the former equation (Eq. 3) was higher than the median eGFR of 74 mL/min/1.73 m^2^ (IQR 64–87 mL/min/1.73 m^2^) in the main analysis using the revised Japanese equation (Eq. 2). Because convergence errors occurred using the former equation (Eq. 3), only results using the latter equation (Ep. 4) are shown in Supplementary Table S6. All HbA1c groups in the prediabetic range were not associated with later onset CKD.

## Discussion

The current analysis of 7176 non-diabetic Japanese residents of Zentsuji city found that all HbA1c groups had a slightly higher point estimate of survival time to CKD onset compared with the normal range (HbA1c 5.0–5.4%). The point estimates of CKD onset were up to 3% shorter in the prediabetic group with HbA1c 5.5–5.9% and up to 1% longer in the prediabetic group with HbA1c 6.0–6.4%, with 95% CIs across 1 in all models. The present study did not support the previous studies^24,39^. As the strengths of the study, we examined the relationship between detailed HbA1c classification and CKD development in a non-diabetic population. Furthermore, we used the maximum of 24.1 years of the longitudinal data collected by the municipal administration, limited to a single ethnic group of middle-aged or older residents in one city. Such a long follow-up period would be sufficient to detect the onset of the early stage of CKD.

In a recent meta-analysis, the results using the ADA classification combining three studies had an aHR for developing CKD of 1.32 (95% CI 1.16–1.50) for prediabetes (HbA1c 5.7–6.4%) compared with the normal range (HbA1c < 5.7%)^24^. By contrast, there was only one study that used the IEC/NICE classifications, which was from the Atherosclerosis Risk in Communities (ARIC) Study of 10,844 individuals^39^. This study reported an aHR of 1.50 (95% CI 1.32–1.70) for prediabetes (HbA1c 6.0–6.4%) using HbA1c < 6.0% as the reference. In both of the ADA and IEC/NICE classifications, we found different patterns from previous studies (see Supplementary Tables S1 and S2). In all HbA1c groups, the point estimates were somewhat low and the 95% CIs were narrow.

Three factors could contribute to this trend. First, the ARIC Study included more high-risk groups, such as black race and a higher BMI. By contrast, our participants tended to be healthier than the ARIC Study population. Because receiving a checkup is not mandatory, participants who have serious health problems, see a doctor regularly, have low health consciousness, or are socially isolated are less likely to receive a checkup. Indeed, the current study showed that participants who did not develop CKD during the follow-up period had more frequent checkups than those who developed CKD (Table 1). Second, the participants of our study may have smaller variability of characteristics. Because the target population of our study was limited to non-diabetic Japanese residents of a single city, variations in characteristics are expected to be less than the participants of the ARIC Study, which was conducted in four U.S. states. Third, the time-varying, period-specific HRs had a built-in selection bias and were influenced by a participant’s changing susceptibility to CKD during the follow-up period^49,50^. Furthermore, the long follow-up period of the study may have further influenced this trend. The above factors may explain the small risk and narrow 95% CIs.

There were several limitations in our study. First, our study is based on a small rural city with a population of only approximately 30,000. Furthermore, our study subjects were recruited through voluntary participation instead of random sampling, which could result in poor generalizability. Second, the exact date of CKD onset was unknown in our study. Furthermore, participants may have experienced multiple states (CKD onset, recovery, and/or recurrence) between observations, particularly if the interval between observations was long. These are limitations of cohort studies using annual checkup data, in which participants can choose whether or not to receive checkups. Third, it was necessary to select the parametric accelerated failure time model instead of the Cox PH model as in previous studies because the data violates the PH assumption^24,39^. This made it difficult to simply compare the results with those of previous studies.

Fourth, as described above, there was a selection bias for participants who were healthier than the general population, which may have led to an overestimation of survival. Using 574,024 Japanese adults who participated in the 2005 health checkup, it was estimated that approximately 13% of the general population in Japan exhibits CKD^51^. As noted above, the prevalence of CKD in our study at study entry was approximately 30%, which is higher than these estimates. Of the excluded participants with prevalent CKD at study entry, 53.8% were aged over 70 years, and 51.3% in the over 70 age category were excluded. Therefore, there were only 21.6% of participants aged ≥ 70 years in the current study. The exclusion of the majority of elderly participants in the current analysis may explain the lower risk compared with previous studies.

Fifth, there was a possibility of measurement error because renal function had to be estimated using the revised Japanese equation (Eq. 2) because there was no definitive physician-diagnosed renal function information. To examine the impact of the estimation method on the results, we further used the 2009 CKD-EPI equation (Eq. 3) and the 2009 CKD-EPI equation modified for the Japanese (Eq. 4) and compared the results to those of the main analysis in the sixth sensitivity analysis (see Supplementary Table S6). Convergence errors were observed using the former equation (Eq. 3), which can be partially explained by the higher median eGFR compared with the main analysis and by the reports of a previous study of Japanese adults without kidney disease: the agreement of CKD classification by the eGFR was low for the former (Eq. 3) and the revised Japanese equation (Eq. 2) and high for the latter (Eq. 4) and the revised Japanese equation (Eq. 2)^52^. Another possible source of measurement error was the failure to measure inulin clearance, the gold standard method for estimating the glomerular filtration rate. This was due to the nature of the annual checkups conducted by the local government, offered to large numbers of people for the purpose of secondary prevention, making such measurements impossible as being uneconomical or too time-consuming. Older age and low muscle mass decrease serum creatinine excretion, resulting in an overestimated renal function, leading to misclassification of CKD^2^. This misclassification led to an underestimation of the results of this present study. In addition to adjusting for age and BMI categories, we performed the third sensitivity analysis stratified by age categories (see Supplementary Table S3). The younger age category of 34–69 years displayed a similar trend to those of the main analysis, but the older age category of ≥ 70 years showed convergence errors, possibly due to the 56.9% shorter total time at risk. Additionally, it must be noted that the change in HbA1c criteria from the JDS (1998–2012) to NGSP (2013–2022) is one of the disadvantages of a long-term follow-up. To mitigate this disadvantage, HbA1c values were standardized using the officially certified equation (Eq. 1)^31^. The fifth sensitivity analysis examined how trends would vary by HbA1c criteria (see Supplementary Table S5). We did not retain sufficient power because the total time at risk was shortened by 51.9% for JDS and 59.7% for NGSP.

Sixth, there may have been unmeasured confounding factors, such as the prevalence of diseases, family history, medication, physical activity and diet, and socioeconomic status among many others. Seventh, unobserved factors such as iron deficiency anemia or pregnancy could have caused a misclassification^53,54^. Eighth, there was a possibility of reverse causation. To address this concern, we conducted the fourth sensitivity analysis and found similar trends to the main analysis (see Supplementary Table S4). There was a slight effect of a longer survival in all HbA1c groups in the prediabetic range compared with the main analysis in Table 4. This implied that prevalent proteinuria with HbA1c in the prediabetic range might shorten survival in this study.

Finally, the impact of competing risks, such as dialysis, kidney transplantation, and death could not be evaluated due to unobserved data. In this study, the competing risk for dialysis was expected to be low because the threshold for CKD prevalence was an eGFR to < 60 mL/min/1.73 m^2^, which was higher than the eGFR criteria for dialysis initiation. Furthermore, according to the latest statistics from the United States Renal Data System for 2020, the incidence of kidney transplantation for all ages in Japan was 14 per million population^55^. This implies that the impact of the competing risk from kidney transplantation was likely to be negligible in the present study, which used the data from a city with approximately 30,000 citizens. However, in the present study, the impact of competing risk from death was not negligible. Because participants who died during the observation period were right-censored and had no risk of subsequent CKD onset, the results of this study may be biased, particularly in older participants^56^.

In conclusion, by analyzing the longitudinal data from a Japanese population of 7176 middle-aged or older participants without diabetes, we found that the prediabetic ranges of HbA1c 5.5–5.9% and 6.0–6.4% had no explicit association with new onset of CKD. Our findings did not support the previous findings that the early stage of prediabetes increases the risk of CKD onset. In a healthier population than the general population without diabetes, risk factors other than HbA1c, such as a BMI ≥ 25 kg/m^2^, may be present that increase the risk of developing subsequent CKD.

### Supplementary Information

Supplementary Tables.

## Data Availability

All data generated or analyzed during this study are included in this published article and its Supplementary Information files.
