# Supplementary material for: A population-based longitudinal study on glycated hemoglobin levels and new-onset chronic kidney disease among non-diabetic Japanese adults
Source: Sci Rep. 2023 Aug 23;13:13770. doi: 10.1038/s41598-023-40300-8 (PMC10447421; doi:10.1038/s41598-023-40300-8)
Supplement: Supplementary file 1 — Supplementary Tables. [file 41598_2023_40300_MOESM1_ESM.docx]

Supplementary information

A population-based longitudinal study on glycated hemoglobin levels and new-onset chronic kidney disease among non-diabetic Japanese adults

# Author affiliations

Yukari Okawa^1^, Etsuji Suzuki^1,2^, Toshiharu Mitsuhashi^3^, Toshihide Tsuda^4^, Takashi Yorifuji^1^

^1^Department of Epidemiology, Graduate School of Medicine, Dentistry and Pharmaceutical Sciences, Okayama University, Okayama, Japan

^2^Department of Epidemiology, Harvard T.H. Chan School of Public Health, Boston, MA, USA

^3^Center for Innovative Clinical Medicine, Okayama University Hospital, Okayama, Japan

^4^Department of Human Ecology, Graduate School of Environmental and Life Science, Okayama University, Okayama, Japan

# Corresponding author

Yukari Okawa

Department of Epidemiology, Graduate School of Medicine, Dentistry and Pharmaceutical Sciences, Okayama University,

2-5-1 Shikata-cho, Kita-ku, Okayama 700-8558, Japan

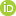
 <https://orcid.org/0000-0002-1704-6609>

Tel. +81 86 223 7151 (ext. 7176)

[okw3923@gmail.com](mailto:okw3923@gmail.com)

| **Supplementary Table S1**. New onset of chronic kidney disease by HbA1c category using the American Diabetes Association (ADA) classification among 7,176 non-diabetic Japanese citizens of Zentsuji city (Total time at risk: 55,604.4 person-years, 1998–2022). | | | | |
| --- | --- | --- | --- | --- |
|  | Model 1 | Model 2 | Model 3 | Model 4 |
| Covariates | aTR (95% CI) | aTR (95% CI) | aTR (95% CI) | aTR (95% CI) |
| HbA1c category | | | | |
| <5.7% (reference) | 1.00 | 1.00 | 1.00 | 1.00 |
| 5.7%–6.4% | 1.00 (0.97–1.02) | 1.00 (0.98–1.03) | 1.00 (0.98–1.02) | 1.00 (0.98–1.02) |
| Sex | | | | |
| Female (reference) | 1.00 | 1.00 | 1.00 | 1.00 |
| Male | 1.00 (0.98–1.02) | 0.99 (0.97–1.02) | 0.99 (0.96–1.02) | 0.99 (0.96–1.02) |
| Age category | | | | |
| 34–59 (reference) | 1.00 | 1.00 | 1.00 | 1.00 |
| 60–69 | 1.02 (0.98–1.06) | 1.02 (0.98–1.06) | 1.02 (0.98–1.06) | 1.02 (0.98–1.06) |
| ≥70 | 1.00 (0.94–1.05) | 1.00 (0.94–1.06) | 1.00 (0.95–1.06) | 1.00 (0.94–1.06) |
| BMI category^a^ | | | | |
| Normal (reference) |  | 1.00 | 1.00 | 1.00 |
| Overweight or obese |  | 0.94 (0.92–0.96) | 0.95 (0.92–0.97) | 0.95 (0.92–0.97) |
| Self-reported drinking status | | | | |
| Nondrinker (reference) |  | 1.00 | 1.00 | 1.00 |
| Drinker |  | 1.01 (0.98–1.03) | 1.01 (0.98–1.04) | 1.01 (0.98–1.04) |
| Self-reported smoking status | | | | |
| Nonsmoker (reference) |  | 1.00 | 1.00 | 1.00 |
| Smoker |  | 1.01 (0.98–1.05) | 1.01 (0.98–1.05) | 1.02 (0.98–1.05) |
| Hypertension^b^ | | | | |
| No (reference) |  |  | 1.00 | 1.00 |
| Yes |  |  | 0.95 (0.93–0.98) | 0.95 (0.93–0.98) |
| Dyslipidemia^c^ | | | | |
| No (reference) |  |  | 1.00 | 1.00 |
| Yes |  |  | 0.99 (0.97–1.02) | 1.00 (0.97–1.02) |
| Residential area | | | | |
| East (reference) |  |  |  | 1.00 |
| Tatsukawa |  |  |  | 1.04 (1.00–1.08) |
| South |  |  |  | 1.02 (0.98–1.06) |
| Fudeoka |  |  |  | 1.01 (0.97–1.06) |
| Center |  |  |  | 0.98 (0.94–1.02) |
| Yoshiwara |  |  |  | 0.98 (0.94–1.02) |
| West |  |  |  | 1.00 (0.96–1.05) |
| Yogita |  |  |  | 1.05 (1.00–1.10) |
| Abbreviations: aTR, adjusted time ratio; CI, confidence interval; HbA1c, glycated hemoglobin. | | | | |
| ^a^ Overweight or obese is defined as a body mass index ≥25 kg/m^2^. | | | | |
| ^b^ Hypertension is defined as systolic blood pressure ≥140 mmHg and/or diastolic blood pressure ≥90 mmHg. | | | | |
| ^c^ Dyslipidemia is defined as serum low density lipoprotein cholesterol ≥140 mg/dL and/or serum high density lipoprotein cholesterol <40 mg/dL. | | | | |
| Multiple imputed variables: overweight or obese, self-reported drinking status, self-reported smoking status, hypertension, dyslipidemia, and residential area. | | | | |
| Model 1: Adjusted for sex and age category. | | | | |
| Model 2: Adjusted for both variables of Model 1, overweight or obese, self-reported drinking status, and self-reported smoking status. | | | | |
| Model 3: Adjusted for all variables of Model 2, hypertension, and dyslipidemia. | | | | |
| Model 4: Adjusted for all variables of Model 3 and residential area. | | | | |

| **Supplementary Table S2**. New onset of chronic kidney disease by HbA1c category using the International Expert Committee (IEC) classification and the National Institute for Health and Care Excellence (NICE) classification among 7,176 non-diabetic Japanese citizens of Zentsuji city (Total time at risk: 55,604.4 person-years, 1998–2022). | | | | |
| --- | --- | --- | --- | --- |
|  | Model 1 | Model 2 | Model 3 | Model 4 |
| Covariates | aTR (95% CI) | aTR (95% CI) | aTR (95% CI) | aTR (95% CI) |
| HbA1c category | | | | |
| <6.0% (reference) | 1.00 | 1.00 | 1.00 | 1.00 |
| 6.0%–6.4% | 1.01 (0.98–1.05) | 1.02 (0.99–1.05) | 1.02 (0.99–1.06) | 1.02 (0.98–1.06) |
| Sex | | | | |
| Female (reference) | 1.00 | 1.00 | 1.00 | 1.00 |
| Male | 1.00 (0.98–1.02) | 0.99 (0.97–1.02) | 0.99 (0.96–1.02) | 0.99 (0.96–1.02) |
| Age category | | | | |
| 34–59 (reference) | 1.00 | 1.00 | 1.00 | 1.00 |
| 60–69 | 1.02 (0.98–1.06) | 1.02 (0.98–1.06) | 1.02 (0.98–1.06) | 1.02 (0.98–1.06) |
| ≥70 | 1.00 (0.94–1.05) | 1.00 (0.94–1.06) | 1.00 (0.95–1.06) | 1.00 (0.94–1.06) |
| BMI category^a^ | | | | |
| Normal (reference) |  | 1.00 | 1.00 | 1.00 |
| Overweight or obese |  | 0.94 (0.92–0.96) | 0.95 (0.92–0.97) | 0.94 (0.92–0.97) |
| Self-reported drinking status | | | | |
| Nondrinker (reference) |  | 1.00 | 1.00 | 1.00 |
| Drinker |  | 1.01 (0.98–1.03) | 1.01 (0.98–1.04) | 1.01 (0.98–1.04) |
| Self-reported smoking status | | | | |
| Nonsmoker (reference) |  | 1.00 | 1.00 | 1.00 |
| Smoker |  | 1.01 (0.98–1.05) | 1.02 (0.98–1.05) | 1.02 (0.98–1.05) |
| Hypertension^b^ | | | | |
| No (reference) |  |  | 1.00 | 1.00 |
| Yes |  |  | 0.95 (0.93–0.98) | 0.95 (0.93–0.98) |
| Dyslipidemia^c^ | | | | |
| No (reference) |  |  | 1.00 | 1.00 |
| Yes |  |  | 0.99 (0.97–1.02) | 0.99 (0.97–1.02) |
| Residential area | | | | |
| East (reference) |  |  |  | 1.00 |
| Tatsukawa |  |  |  | 1.04 (1.00–1.08) |
| South |  |  |  | 1.02 (0.98–1.06) |
| Fudeoka |  |  |  | 1.01 (0.97–1.06) |
| Center |  |  |  | 0.98 (0.94–1.02) |
| Yoshiwara |  |  |  | 0.98 (0.94–1.02) |
| West |  |  |  | 1.00 (0.96–1.05) |
| Yogita |  |  |  | 1.05 (1.00–1.10) |
| Abbreviations: aTR, adjusted time ratio; CI, confidence interval; HbA1c, glycated hemoglobin. | | | | |
| ^a^ Overweight or obese is defined as a body mass index ≥25 kg/m^2^. | | | | |
| ^b^ Hypertension is defined as systolic blood pressure ≥140 mmHg and/or diastolic blood pressure ≥90 mmHg. | | | | |
| ^c^ Dyslipidemia is defined as serum low density lipoprotein cholesterol ≥140 mg/dL and/or serum high density lipoprotein cholesterol <40 mg/dL. | | | | |
| Multiple imputed variables: overweight or obese, self-reported drinking status, self-reported smoking status, hypertension, dyslipidemia, and residential area. | | | | |
| Model 1: Adjusted for sex and age category. | | | | |
| Model 2: Adjusted for both variables of Model 1, overweight or obese, self-reported drinking status, and self-reported smoking status. | | | | |
| Model 3: Adjusted for all variables of Model 2, hypertension, and dyslipidemia. | | | | |
| Model 4: Adjusted for all variables of Model 3 and residential area. | | | | |

| **Supplementary Table S3**. New onset of chronic kidney disease by HbA1c category among 5,260 non-diabetic Japanese citizens aged 34–69 years in Zentsuji city (Total time at risk: 32,243.6 person-years, 1998–2022). | | | | |
| --- | --- | --- | --- | --- |
|  | Model 1 | Model 2 | Model 3 | Model 4 |
| Covariates | aTR (95% CI) | aTR (95% CI) | aTR (95% CI) | aTR (95% CI) |
| HbA1c category | | | | |
| <5.0% | 1.13 (1.02–1.25) | 1.12 (1.02–1.24) | 1.13 (1.02–1.26) | 1.13 (1.02–1.26) |
| 5.0%–5.4% (reference) | 1.00 | 1.00 | 1.00 | 1.00 |
| 5.5%–5.9% | 0.96 (0.92–1.01) | 0.97 (0.93–1.02) | 0.97 (0.92–1.01) | 0.96 (0.92–1.01) |
| 6.0%–6.4% | 0.99 (0.92–1.07) | 1.01 (0.93–1.08) | 1.00 (0.93–1.08) | 1.00 (0.92–1.08) |
| Sex | | | | |
| Female (reference) | 1.00 | 1.00 | 1.00 | 1.00 |
| Male | 0.99 (0.95–1.03) | 0.97 (0.92–1.02) | 0.97 (0.92–1.02) | 0.97 (0.92–1.02) |
| Age category | | | | |
| 34–59 (reference) | 1.00 | 1.00 | 1.00 | 1.00 |
| 60–69 | 0.94 (0.87–1.01) | 0.94 (0.87–1.01) | 0.94 (0.87–1.01) | 0.94 (0.87–1.01) |
| ≥70 | – | – | – | – |
| BMI category^a^ | | | | |
| Normal (reference) |  | 1.00 | 1.00 | 1.00 |
| Overweight or obese |  | 0.93 (0.88–0.98) | 0.94 (0.89–0.99) | 0.94 (0.89–0.99) |
| Self-reported drinking status | | | | |
| Nondrinker (reference) |  | 1.00 | 1.00 | 1.00 |
| Drinker |  | 1.00 (0.95–1.06) | 1.01 (0.96–1.06) | 1.01 (0.95–1.06) |
| Self-reported smoking status | | | | |
| Nonsmoker (reference) |  | 1.00 | 1.00 | 1.00 |
| Smoker |  | 1.09 (1.01–1.17) | 1.09 (1.00–1.18) | 1.09 (1.01–1.18) |
| Hypertension^b^ | | | | |
| No (reference) |  |  | 1.00 | 1.00 |
| Yes |  |  | 0.91 (0.86–0.96) | 0.91 (0.86–0.96) |
| Dyslipidemia^c^ | | | | |
| No (reference) |  |  | 1.00 | 1.00 |
| Yes |  |  | 1.01 (0.96–1.05) | 1.01 (0.97–1.06) |
| Residential area | | | | |
| East (reference) |  |  |  | 1.00 |
| Tatsukawa |  |  |  | 1.09 (1.00–1.18) |
| South |  |  |  | 1.05 (0.98–1.14) |
| Fudeoka |  |  |  | 1.04 (0.96–1.13) |
| Center |  |  |  | 0.94 (0.87–1.02) |
| Yoshiwara |  |  |  | 0.94 (0.87–1.01) |
| West |  |  |  | 1.04 (0.95–1.14) |
| Yogita |  |  |  | 1.09 (0.98–1.20) |
| Abbreviations: aTR, adjusted time ratio; CI, confidence interval; HbA1c, glycated hemoglobin. | | | | |
| ^a^ Overweight or obese is defined as a body mass index ≥25 kg/m^2^. | | | | |
| ^b^ Hypertension is defined as systolic blood pressure ≥140 mmHg and/or diastolic blood pressure ≥90 mmHg. | | | | |
| ^c^ Dyslipidemia is defined as serum low density lipoprotein cholesterol ≥140 mg/dL and/or serum high density lipoprotein cholesterol <40 mg/dL. | | | | |
| Multiple imputed variables: overweight or obese, self-reported drinking status, self-reported smoking status, hypertension, dyslipidemia, and residential area. | | | | |
| Model 1: Adjusted for sex and age category. | | | | |
| Model 2: Adjusted for both variables of Model 1, overweight or obese, self-reported drinking status, and self-reported smoking status. | | | | |
| Model 3: Adjusted for all variables of Model 2, hypertension, and dyslipidemia. | | | | |
| Model 4: Adjusted for all variables of Model 3 and residential area. | | | | |

| **Supplementary Table S4**. New onset of chronic kidney disease by HbA1c category among 6,880 non-diabetic Japanese citizens of Zentsuji city excluding those with missing data of a urine dipstick test or with a proteinuria ≥+1 at study entry (Total time at risk: 53,250.7 person-years, 1998–2022). | | | | |
| --- | --- | --- | --- | --- |
|  | Model 1 | Model 2 | Model 3 | Model 4 |
| Covariates | aTR (95% CI) | aTR (95% CI) | aTR (95% CI) | aTR (95% CI) |
| HbA1c category | | | | |
| <5.0% | 1.24 (1.10–1.40) | 1.24 (1.10–1.40) | 1.25 (1.10–1.41) | 1.25 (1.10–1.41) |
| 5.0%–5.4% (reference) | 1.00 | 1.00 | 1.00 | 1.00 |
| 5.5%–5.9% | 0.99 (0.93–1.04) | 0.99 (0.94–1.05) | 0.99 (0.94–1.05) | 0.99 (0.94–1.05) |
| 6.0%–6.4% | 1.01 (0.91–1.13) | 1.02 (0.92–1.14) | 1.02 (0.92–1.15) | 1.02 (0.92–1.14) |
| Sex | | | | |
| Female (reference) | 1.00 | 1.00 | 1.00 | 1.00 |
| Male | 1.00 (0.98–1.02) | 0.99 (0.97–1.02) | 0.99 (0.97–1.02) | 0.99 (0.96–1.02) |
| Age category | | | | |
| 34–59 (reference) | 1.00 | 1.00 | 1.00 | 1.00 |
| 60–69 | 1.04 (0.98–1.11) | 1.05 (0.99–1.11) | 1.05 (0.99–1.11) | 1.05 (0.99–1.11) |
| ≥70 | 0.99 (0.93–1.07) | 1.00 (0.93–1.07) | 1.00 (0.93–1.08) | 1.00 (0.93–1.07) |
| BMI category^a^ | | | | |
| Normal (reference) |  | 1.00 | 1.00 | 1.00 |
| Overweight or obese |  | 0.94 (0.91–0.96) | 0.95 (0.92–0.97) | 0.94 (0.92–0.97) |
| Self-reported drinking status | | | | |
| Nondrinker (reference) |  | 1.00 | 1.00 | 1.00 |
| Drinker |  | 1.01 (0.98–1.04) | 1.01 (0.98–1.04) | 1.01 (0.98–1.04) |
| Self-reported smoking status | | | | |
| Nonsmoker (reference) |  | 1.00 | 1.00 | 1.00 |
| Smoker |  | 1.01 (0.97–1.05) | 1.01 (0.97–1.05) | 1.01 (0.97–1.05) |
| Hypertension^b^ | | | | |
| No (reference) |  |  | 1.00 | 1.00 |
| Yes |  |  | 0.94 (0.92–0.97) | 0.94 (0.92–0.97) |
| Dyslipidemia^c^ | | | | |
| No (reference) |  |  | 1.00 | 1.00 |
| Yes |  |  | 0.98 (0.96–1.01) | 0.99 (0.96–1.01) |
| Residential area | | | | |
| East (reference) |  |  |  | 1.00 |
| Tatsukawa |  |  |  | 1.06 (1.02–1.10) |
| South |  |  |  | 1.02 (0.98–1.06) |
| Fudeoka |  |  |  | 1.02 (0.98–1.07) |
| Center |  |  |  | 0.98 (0.94–1.02) |
| Yoshiwara |  |  |  | 0.99 (0.94–1.04) |
| West |  |  |  | 1.00 (0.96–1.05) |
| Yogita |  |  |  | 1.05 (1.00–1.11) |
| Interaction | | | | |
| HbA1c category × age category | | | | |
| (reference: HbA1c 5.0%–5.4%, 34–59 years) | | | | |
| HbA1c <5.0% × age 60–69 | 0.80 (0.68–0.93) | 0.80 (0.69–0.93) | 0.80 (0.68–0.93) | 0.80 (0.68–0.93) |
| HbA1c <5.0% × age ≥70 | 0.83 (0.72–0.96) | 0.83 (0.72–0.96) | 0.83 (0.72–0.96) | 0.83 (0.72–0.96) |
| HbA1c 5.5%–5.9% × age 60–69 | 0.98 (0.91–1.05) | 0.98 (0.91–1.05) | 0.97 (0.91–1.05) | 0.97 (0.90–1.05) |
| HbA1c 5.5%–5.9% × age ≥70 | 1.02 (0.95–1.09) | 1.02 (0.95–1.09) | 1.02 (0.95–1.09) | 1.02 (0.95–1.09) |
| HbA1c 6.0%–6.4% × age 60–69 | 0.98 (0.87–1.11) | 0.98 (0.87–1.11) | 0.98 (0.86–1.11) | 0.98 (0.86–1.11) |
| HbA1c 6.0%–6.4% × age ≥70 | 1.02 (0.90–1.15) | 1.02 (0.90–1.15) | 1.02 (0.90–1.15) | 1.02 (0.90–1.16) |
| Abbreviations: aTR, adjusted time ratio; CI, confidence interval; HbA1c, glycated hemoglobin. | | | | |
| ^a^ Overweight or obese is defined as a body mass index ≥25 kg/m^2^. | | | | |
| ^b^ Hypertension is defined as systolic blood pressure ≥140 mmHg and/or diastolic blood pressure ≥90 mmHg. | | | | |
| ^c^ Dyslipidemia is defined as serum low density lipoprotein cholesterol ≥140 mg/dL and/or serum high density lipoprotein cholesterol <40 mg/dL. | | | | |
| Multiple imputed variables: overweight or obese, self-reported drinking status, self-reported smoking status, hypertension, dyslipidemia, and residential area. | | | | |
| Model 1: Adjusted for sex and age category. | | | | |
| Model 2: Adjusted for both variables of Model 1, overweight or obese, self-reported drinking status, and self-reported smoking status. | | | | |
| Model 3: Adjusted for all variables of Model 2, hypertension, and dyslipidemia. | | | | |
| Model 4: Adjusted for all variables of Model 3 and residential area. | | | | |

| **Supplementary Table S5**. New onset of chronic kidney disease by HbA1c category among 7,161 non-diabetic Japanese citizens of Zentsuji city stratified by HbA1c unit (Total time at risk: 26,765.1 person-years for JDS and 22,430.1 person-years for NGSP). | | | | | | | | |
| --- | --- | --- | --- | --- | --- | --- | --- | --- |
|  | HbA1c unit (year, N)^a^ | | | | | | | |
|  | JDS (1998–2012, n=5,103) | | | | NGSP (2013–2022, N=4,879) | | | |
|  | Model 1 | Model 2 | Model 3 | Model 4 | Model 1 | Model 2 | Model 3 | Model 4 |
| Covariates | aTR (95% CI) | aTR (95% CI) | aTR (95% CI) | aTR (95% CI) | aTR (95% CI) | aTR (95% CI) | aTR (95% CI) | aTR (95% CI) |
| HbA1c category | | | | | | | | |
| <5.0% | 1.07 (1.02–1.12) | 1.06 (1.01–1.12) | 1.07 (1.02–1.13) | 1.07 (1.02–1.13) | 7.98 (6.13–10.39) | 8.17 (6.27–10.66) | 8.22 (6.29–10.73) | 7.37 (5.69–9.55) |
| 5.0%–5.4% (reference) | 1.00 | 1.00 | 1.00 | 1.00 | 1.00 | 1.00 | 1.00 | 1.00 |
| 5.5%–5.9% | 1.00 (0.95–1.06) | 1.01 (0.95–1.06) | 1.01 (0.95–1.07) | 1.01 (0.96–1.07) | 1.00 (0.94–1.07) | 1.00 (0.94–1.07) | 1.00 (0.94–1.07) | 1.00 (0.94–1.07) |
| 6.0%–6.4% | 1.20 (1.04–1.39) | 1.21 (1.04–1.40) | 1.23 (1.05–1.44) | 1.22 (1.05–1.43) | 1.05 (0.92–1.19) | 1.05 (0.93–1.19) | 1.05 (0.93–1.20) | 1.05 (0.93–1.20) |
| Sex | | | | | | | | |
| Female (reference) | 1.00 | 1.00 | 1.00 | 1.00 | 1.00 | 1.00 | 1.00 | 1.00 |
| Male | 1.02 (0.98–1.06) | 1.01 (0.96–1.06) | 1.01 (0.95–1.06) | 1.01 (0.95–1.06) | 0.98 (0.97–1.00) | 0.99 (0.97–1.01) | 0.99 (0.97–1.01) | 0.99 (0.97–1.00) |
| Age category | | | | | | | | |
| 34–59 (reference) | 1.00 | 1.00 | 1.00 | 1.00 | 1.00 | 1.00 | 1.00 | 1.00 |
| 60–69 | 1.03 (0.96–1.10) | 1.03 (0.96–1.10) | 1.03 (0.95–1.10) | 1.03 (0.95–1.10) | 1.08 (1.02–1.15) | 1.08 (1.02–1.15) | 1.08 (1.02–1.15) | 1.08 (1.02–1.15) |
| ≥70 | 0.95 (0.84–1.07) | 0.95 (0.84–1.07) | 0.95 (0.84–1.07) | 0.95 (0.84–1.07) | 1.13 (1.06–1.21) | 1.14 (1.07–1.21) | 1.14 (1.07–1.21) | 1.14 (1.07–1.21) |
| BMI category^a^ | | | | | | | | |
| Normal (reference) |  | 1.00 | 1.00 | 1.00 |  | 1.00 | 1.00 | 1.00 |
| Overweight or obese |  | 0.95 (0.91–1.00) | 0.97 (0.92–1.01) | 0.96 (0.92–1.01) |  | 0.96 (0.94–0.98) | 0.96 (0.94–0.98) | 0.96 (0.94–0.98) |
| Self-reported drinking status | | | | | | | | |
| Nondrinker (reference) |  | 1.00 | 1.00 | 1.00 |  | 1.00 | 1.00 | 1.00 |
| Drinker |  | 1.00 (0.95–1.06) | 1.01 (0.96–1.07) | 1.01 (0.96–1.07) |  | 0.99 (0.97–1.01) | 0.99 (0.97–1.01) | 0.99 (0.97–1.01) |
| Self-reported smoking status | | | | | | | | |
| Nonsmoker (reference) |  | 1.00 | 1.00 | 1.00 |  | 1.00 | 1.00 | 1.00 |
| Smoker |  | 1.03 (0.96–1.10) | 1.03 (0.96–1.11) | 1.03 (0.96–1.11) |  | 1.01 (0.98–1.04) | 1.01 (0.98–1.04) | 1.01 (0.98–1.04) |
| Hypertension^c^ | | | | | | | | |
| No (reference) |  |  | 1.00 | 1.00 |  |  | 1.00 | 1.00 |
| Yes |  |  | 0.92 (0.88–0.97) | 0.92 (0.87–0.97) |  |  | 0.99 (0.97–1.00) | 0.99 (0.97–1.00) |
| Dyslipidemia^d^ | | | | | | | | |
| No (reference) |  |  | 1.00 | 1.00 |  |  | 1.00 | 1.00 |
| Yes |  |  | 1.01 (0.96–1.05) | 1.01 (0.96–1.05) |  |  | 1.00 (0.98–1.01) | 1.00 (0.98–1.01) |
| Residential area | | | | | | | | |
| East (reference) |  |  |  | 1.00 |  |  |  | 1.00 |
| Tatsukawa |  |  |  | 1.06 (0.98–1.14) |  |  |  | 0.99 (0.96–1.02) |
| South |  |  |  | 1.05 (0.98–1.13) |  |  |  | 0.98 (0.96–1.01) |
| Fudeoka |  |  |  | 0.96 (0.89–1.04) |  |  |  | 1.03 (0.99–1.06) |
| Center |  |  |  | 0.98 (0.91–1.06) |  |  |  | 0.98 (0.95–1.01) |
| Yoshiwara |  |  |  | 0.95 (0.88–1.03) |  |  |  | 0.98 (0.95–1.01) |
| West |  |  |  | 1.01 (0.93–1.10) |  |  |  | 0.97 (0.94–1.00) |
| Yogita |  |  |  | 1.12 (1.02–1.23) |  |  |  | 0.99 (0.95–1.03) |
| Interaction | | | | | | | | |
| HbA1c category × age category | | | | | | | | |
| (reference: HbA1c 5.0%–5.4%, 34–59 years) | | | | | | | | |
| HbA1c <5.0% × age 60–69 | – | – | – | – | 0.12 (0.09–0.16) | 0.12 (0.09–0.16) | 0.12 (0.09–0.16) | 0.13 (0.10–0.18) |
| HbA1c <5.0% × age ≥70 | – | – | – | – | 0.12 (0.09–0.16) | 0.12 (0.09–0.16) | 0.12 (0.09–0.16) | 0.13 (0.10–0.18) |
| HbA1c 5.5%–5.9% × age 60–69 | – | – | – | – | 1.01 (0.93–1.09) | 1.01 (0.93–1.09) | 1.01 (0.93–1.09) | 1.01 (0.93–1.09) |
| HbA1c 5.5%–5.9% × age ≥70 | – | – | – | – | 1.00 (0.93–1.07) | 1.00 (0.93–1.07) | 1.00 (0.93–1.07) | 1.00 (0.93–1.07) |
| HbA1c 6.0%–6.4% × age 60–69 | – | – | – | – | 0.98 (0.85–1.13) | 0.99 (0.86–1.13) | 0.98 (0.86–1.13) | 0.99 (0.86–1.13) |
| HbA1c 6.0%–6.4% × age ≥70 | – | – | – | – | 0.97 (0.85–1.11) | 0.97 (0.85–1.11) | 0.97 (0.85–1.11) | 0.97 (0.85–1.11) |
| Abbreviations: aTR, adjusted time ratio; CI, confidence interval; HbA1c, glycated hemoglobin; JDS, Japan Diabetes Society; NGSP, National Glycohemoglobin Standardization Program. | | | | | | | | |
| ^a^ Each HbA1c unit contains the same participants. | | | | | | | | |
| ^b^ Overweight or obese is defined as a body mass index ≥25 kg/m^2^. | | | | | | | | |
| ^c^ Hypertension is defined as systolic blood pressure ≥140 mmHg and/or diastolic blood pressure ≥90 mmHg. | | | | | | | | |
| ^d^ Dyslipidemia is defined as serum low density lipoprotein cholesterol ≥140 mg/dL and/or serum high density lipoprotein cholesterol <40 mg/dL. | | | | | | | | |
| Multiple imputed variables for JDS: overweight or obese, self-reported drinking status, self-reported smoking status, hypertension, dyslipidemia, and residential area. | | | | | | | | |
| Multiple imputed variables for NGSP: overweight or obese, self-reported drinking status, self-reported smoking status, and residential area. | | | | | | | | |
| Model 1: Adjusted for sex and age category. | | | | | | | | |
| Model 2: Adjusted for both variables of Model 1, overweight or obese, self-reported drinking status, and self-reported smoking status. | | | | | | | | |
| Model 3: Adjusted for all variables of Model 2, hypertension, and dyslipidemia. | | | | | | | | |
| Model 4: Adjusted for all variables of Model 3 and residential area. | | | | | | | | |

| **Supplementary Table S6**. New onset of chronic kidney disease by HbA1c category among 8,484 non-diabetic Japanese citizens of Zentsuji city where eGFR was calculated with the 2009 CKD Epidemiology Collaboration (CKD-EPI) equation (Total time at risk: 73,821.5 person-years, 1998–2022). | | | | |
| --- | --- | --- | --- | --- |
|  | Model 1 | Model 2 | Model 3 | Model 4 |
| Covariates | aTR (95% CI) | aTR (95% CI) | aTR (95% CI) | aTR (95% CI) |
| HbA1c category | | | | |
| <5.0% | 1.05 (1.01–1.09) | 1.05 (1.01–1.09) | 1.05 (1.01–1.10) | 1.05 (1.01–1.10) |
| 5.0%–5.4% (reference) | 1.00 | 1.00 | 1.00 | 1.00 |
| 5.5%–5.9% | 0.99 (0.97–1.01) | 0.99 (0.98–1.01) | 0.99 (0.98–1.01) | 0.99 (0.97–1.01) |
| 6.0%–6.4% | 0.99 (0.97–1.02) | 1.00 (0.97–1.03) | 1.00 (0.97–1.03) | 1.00 (0.97–1.03) |
| Sex | | | | |
| Female (reference) | 1.00 | 1.00 | 1.00 | 1.00 |
| Male | 0.97 (0.95–0.99) | 0.96 (0.94–0.98) | 0.96 (0.94–0.98) | 0.96 (0.94–0.98) |
| Age category | | | | |
| 34–59 (reference) | 1.00 | 1.00 | 1.00 | 1.00 |
| 60–69 | 1.05 (1.02–1.09) | 1.06 (1.02–1.09) | 1.06 (1.03–1.09) | 1.06 (1.03–1.09) |
| ≥70 | 1.01 (0.96–1.05) | 1.01 (0.97–1.05) | 1.01 (0.97–1.05) | 1.01 (0.97–1.05) |
| BMI category^a^ | | | | |
| Normal (reference) |  | 1.00 | 1.00 | 1.00 |
| Overweight or obese |  | 0.95 (0.94–0.97) | 0.96 (0.94–0.98) | 0.96 (0.94–0.98) |
| Self-reported drinking status | | | | |
| Nondrinker (reference) |  | 1.00 | 1.00 | 1.00 |
| Drinker |  | 1.02 (1.00–1.04) | 1.02 (1.00–1.04) | 1.02 (1.00–1.04) |
| Self-reported smoking status | | | | |
| Nonsmoker (reference) |  | 1.00 | 1.00 | 1.00 |
| Smoker |  | 1.00 (0.98–1.03) | 1.00 (0.98–1.03) | 1.00 (0.98–1.03) |
| Hypertension^b^ | | | | |
| No (reference) |  |  | 1.00 | 1.00 |
| Yes |  |  | 0.95 (0.93–0.97) | 0.95 (0.93–0.97) |
| Dyslipidemia^c^ | | | | |
| No (reference) |  |  | 1.00 | 1.00 |
| Yes |  |  | 0.99 (0.97–1.01) | 0.99 (0.97–1.01) |
| Residential area | | | | |
| East (reference) |  |  |  | 1.00 |
| Tatsukawa |  |  |  | 1.01 (0.98–1.04) |
| South |  |  |  | 0.99 (0.96–1.02) |
| Fudeoka |  |  |  | 0.99 (0.96–1.02) |
| Center |  |  |  | 0.97 (0.94–0.99) |
| Yoshiwara |  |  |  | 1.00 (0.97–1.04) |
| West |  |  |  | 1.00 (0.97–1.03) |
| Yogita |  |  |  | 1.00 (0.97–1.04) |
| Abbreviations: aTR, adjusted time ratio; CI, confidence interval; HbA1c, glycated hemoglobin. | | | | |
| ^a^ Overweight or obese is defined as a body mass index ≥25 kg/m^2^. | | | | |
| ^b^ Hypertension is defined as systolic blood pressure ≥140 mmHg and/or diastolic blood pressure ≥90 mmHg. | | | | |
| ^c^ Dyslipidemia is defined as serum low density lipoprotein cholesterol ≥140 mg/dL and/or serum high density lipoprotein cholesterol <40 mg/dL. | | | | |
| Multiple imputed variables: overweight or obese, self-reported drinking status, self-reported smoking status, hypertension, dyslipidemia, and residential area. | | | | |
| Model 1: Adjusted for sex and age category. | | | | |
| Model 2: Adjusted for both variables of Model 1, overweight or obese, self-reported drinking status, and self-reported smoking status. | | | | |
| Model 3: Adjusted for all variables of Model 2, hypertension, and dyslipidemia. | | | | |
| Model 4: Adjusted for all variables of Model 3 and residential area. | | | | |
